# Supplementary figures and images for: Predictive factors affecting percutaneous drainage duration in the percutaneous treatment of common bile duct stones
Source: PLoS One. 2021 Mar 2;16(3):e0248003. doi: 10.1371/journal.pone.0248003 (PMC7924786; doi:10.1371/journal.pone.0248003)

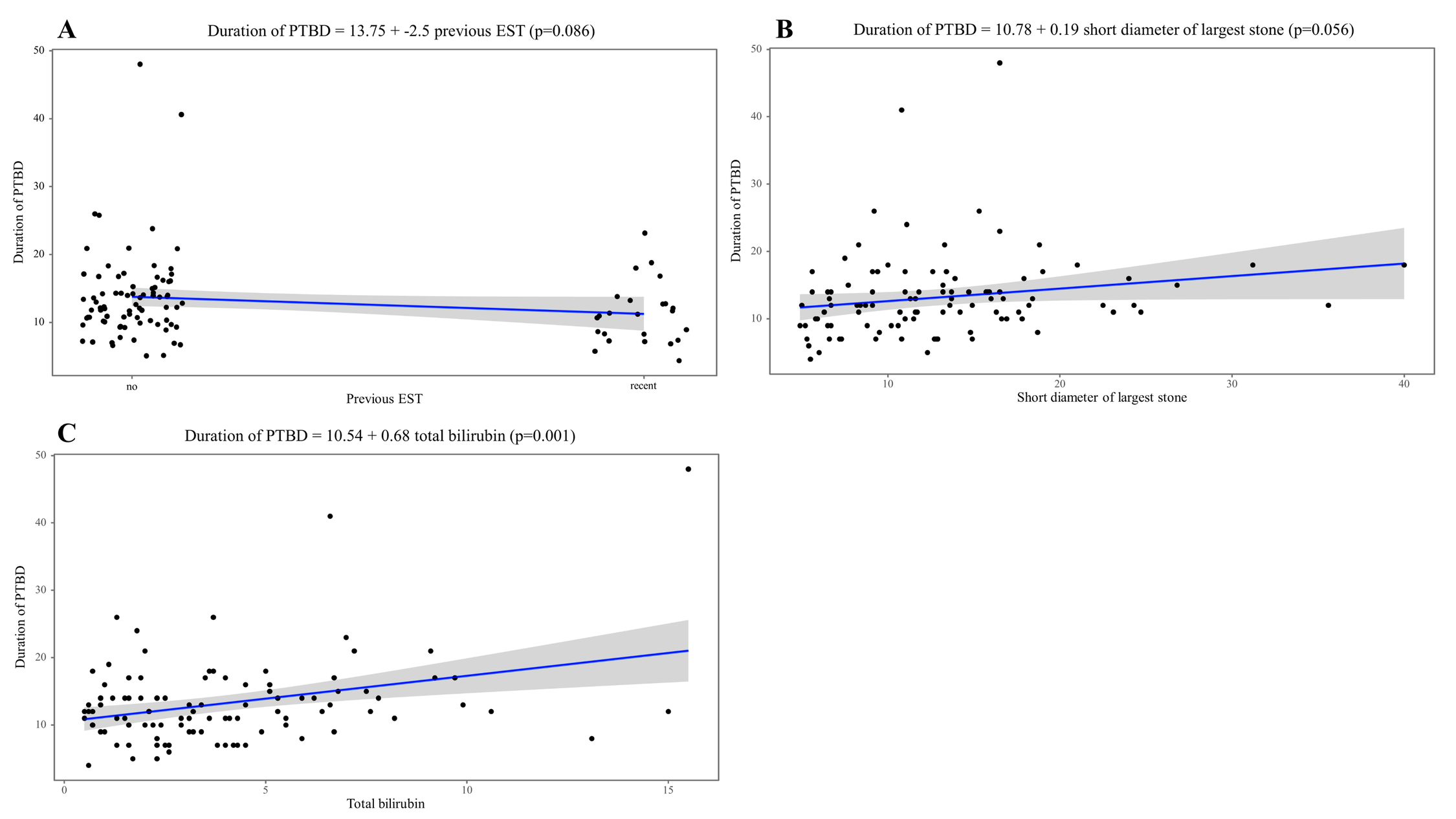

Supplement: S1 Fig — A, previous EST (coefficient = -2.50, P = .086). B, short diameter of the largest stone (coefficient = 0.19, P = .056). C, total bilirubin level (coefficient = 0.68, P < .001). (TIF) [file pone.0248003.s001.tif]
