# Supplementary material for: Predictive factors affecting percutaneous drainage duration in the percutaneous treatment of common bile duct stones
Source: PLoS One. 2021 Mar 2;16(3):e0248003. doi: 10.1371/journal.pone.0248003 (PMC7924786; doi:10.1371/journal.pone.0248003)
Supplement: Attachment — Submitted filename: Response to Reviewers_1228.docx [file pone.0248003.s002.docx]

**December 26, 2020**

**Title: Predictive factors affecting percutaneous drainage duration in the percutaneous treatment of common bile duct stones**

PONE-D-20-31996

**Dear Editor-in-Chief**

I would like to thank you for your letter regarding the revision of our manuscript that was submitted to PLOS ONE.

I appreciate your valuable comments and suggestions. We have revised the manuscript as you suggested, and the recommended changes are in underlined, blue color text. They are as follows.

**Additional Editor Comments:**
1. Why the authors used all variables with P < .20 ? Please explain the rationale with citation.

Model minimization was performed by stepwise backward elimination method. To rule out confounding factors more effectively, we chose P < 0.2 to identify as many potential predictors and confounders as possible at the cost of multiple testing. (Maldonado G, Greenland S. Simulation study of confounder-selection strategies. Am J Epidemiol. 1993;138: 923-936. DOI: 10.1093/oxfordjournals.aje.a116813.)

We revised and added the phrase as follows, in “Methods”

🡺 “…multivariate linear regression analysis with backwards stepwise elimination method was applied, incorporating significant predictive factors identified in the univariate regression analysis”

2. The authors reported Coeff(B) and Std Coeff(β) in table 3and 4. Why the authors reported both? What is the differences these two methods?
In addition, the authors did not mention the Coeff(B) and Std Coeff(β) in method. Please explain in the methods.

Thank you for your comment. Various independent models measured in different units were involved in our multiple regression model. Therefore, we indicated the standard coefficient in order to clarify which of the independent variables measured in different units had a greater effect on PTBD duration.

We revised and added the phrase as follows, in “Methods”

🡺 “The coefficient and the standardized coefficient were indicated in order to clarify which of the independent variables measured in different units had a greater effect on PTBD duration.”

**Journal Requirements:**

1. Please ensure that your manuscript meets PLOS ONE's style requirements, including those for file naming.

We checked the “The PLOS ONE style templates” and revised them to "PLOS ONE's style requirements".

2. Thank you for stating in the text of your manuscript "the requirement for informed patient consent was waived". Please also add this information to your ethics statement in the online submission form.

We checked and added our ethics statement in the online submission form.

3. Please provide more information on data extraction and collection. Please provide:
a) the date the data was obtained by researchers
b) any inclusion criteria used to include patients (we note that you already state exclusion criteria)
c) a list of the data extracted from medical records

As you suggested, we revised and added the phrase as follows, in “Methods”

**🡺 Study design and data collection**

The institutional review board of Seoul Metropolitan Government-Seoul National University Boramae Medical Center approved this retrospective study (registration number; 10-2019-71), and the requirement for informed patient consent was waived. All procedures followed were in accordance with the ethical standards of the responsible committees on human experimentation (institutional and national) and with the Helsinki Declaration of 1964 and later versions. Medical records of consecutive adult hospitalized patients who underwent percutaneous treatment of CBD stones between June 2009 and June 2019 were collected from the electronic medical record database and picture archiving and communication system in our institution. Data such as laboratory blood tests, patient demographics, imaging findings, and treatment history were extracted from medical records. Among the patients whose CBD stone was identified through ERCP or CT images during this period, patients who received percutaneous treatment of CBD stone were finally included in the study. Patients with underlying hepatobiliary malignant tumors were excluded. The data were obtained and consolidated in November 2019.

4. We note that you have indicated that data from this study are available upon request. PLOS only allows data to be available upon request if there are legal or ethical restrictions on sharing data publicly.

In your revised cover letter, please address the following prompts:

a) If there are ethical or legal restrictions on sharing a de-identified data set, please explain them in detail (e.g., data contain potentially identifying or sensitive patient information) and who has imposed them (e.g., an ethics committee). Please also provide contact information for a data access committee, ethics committee, or other institutional body to which data requests may be sent.

b) If there are no restrictions, please upload the minimal anonymized data set necessary to replicate your study findings as either Supporting Information files or to a stable, public repository and provide us with the relevant URLs, DOIs, or accession numbers. Please see <http://www.bmj.com/content/340/bmj.c181.long> for guidelines on how to de-identify and prepare clinical data for publication. For a list of acceptable repositories, please see <http://journals.plos.org/plosone/s/data-availability#loc-recommended-repositories>.

We will update your Data Availability statement on your behalf to reflect the information you provide.

**🡺** The institutional review board (IRB) of “Seoul Metropolitan Government-Seoul National University Boramae Medical Center” approved this retrospective study, and the requirement for informed patient consent was waived.

All data are anonymized and managed but include indirect identifiers (information sources) such as patients' age, sex, treatment dates, and treatment methods. The IRB of our institution does not allow disclosure of such data. The contact information of our IRB center is as follows.

e-mail: [brmirb02734@gmail.com](mailto:brmirb02734@gmail.com)

Tel: +82-2-870-1851

The cover letter was revised to include this information.

5. Your ethics statement should only appear in the Methods section of your manuscript. If your ethics statement is written in any section besides the Methods, please delete it from any other section.

It is confirmed that it is expressed only in the "Methods section".

6. Please include your tables as part of your main manuscript and remove the individual files. Please note that supplementary tables should be uploaded as separate "supporting information" files.

Individual files were removed and tables were included in the main manuscript.

**Reviewer #1:** 

1. What is the reason to choose P < .20 for including variables in univariate regression analysis into multivariate regression analysis for the context of this research topic? Is there any reference for it? Please provide

Model minimization was performed by stepwise backward elimination method. To rule out confounding more effectively, we chose P < 0.2 to identify as many potential predictors and confounders as possible at the cost of multiple testing. (Maldonado G, Greenland S. Simulation study of confounder-selection strategies. Am J Epidemiol. 1993;138:923-936. DOI: 10.1093/oxfordjournals.aje.a116813.)

We revised and added the phrase as follows, in “Methods”

🡺 “…multivariate linear regression analysis with backwards stepwise elimination method was applied, incorporating significant predictive factors identified in the univariate regression analysis”

2. Why despite variable EST not significant in the univariate analysis was included into multivariate analysis? Is there any theoretical base for it? Please provide

As described above, P < 0.2 was accepted as significant in the selection of the risk factors.

**Reviewer #2:**

1. Was a correlation analysis done between the continuous variables and duration of drainage by scatter plot? This is not clear from your methodology section. You need to describe in detail how the univariate analysis was arrived at.

We used linear regression analysis to examine a correlation between the continuous variables and duration of drainage (Pearson’s correlation coefficient test). We added scatter plots of correlated factors (initial total bilirubin level, the short diameter of the largest stone, previous EST) as supplementary tables and the phrase as follows, in “Methods”

🡺 “Correlations were calculated using Pearson’s correlation coefficient test.”

2. What is the take home message from the study based on your findings? Currently you mention it as a factor to decide the choice of treatment; however you also mention that percutaneous drainage is alrady a second line option. SO what is the implication of your paper?

The “treatment plan” mentioned in the conclusion implies not only to the treatment of CBD stones, but also to the overall management of patient care (such as management of associated underlying diseases).

To clarify the above points, we revised and added the phrase as follows, as the last sentence of “discussion” and conclusion of “abstract”

🡺 “Careful assessment of these factors might help the prediction of the treatment period, thereby improving the quality of patient care.”
